# Metabolic syndrome and surgical complications: a systematic review and meta-analysis of 13 million individuals

**DOI:** 10.1097/JS9.0000000000000834

**Published:** 2023-11-01

**Authors:** Philip Norris, Jeff Gow, Thomas Arthur, Aaron Conway, Fergal J. Fleming, Nicholas Ralph

**Affiliations:** a School of Nursing and Midwifery, University of Southern Queensland, Australia; b School of Commerce, University of Southern Queensland, Toowoomba, Australia; c Department of Surgery and Adjunct Professor, Toowoomba Hospital, Centre for Health Research, University of Southern Queensland, Toowoomba, Australia; d University of Sunshine Coast, Moreton Bay, Australia; e Peter Munk Cardiac Centre, University Health Network, Toronto, Canada, Lawrence S. Bloomberg Faculty of Nursing, University of Toronto, Toronto, Canada; f University of Rochester Medical Center, USA; g Senior Research Associate, School of Accounting, Economics and Finance, University of KwaZulu- Natal, Durban, South Africa

**Keywords:** metabolic syndrome, complications, surgical procedure, operative, adverse events

## Abstract

**Background:**

Metabolic syndrome (MetS) is characterised by the presence of at least three of the five following components: insulin resistance, obesity, chronic hypertension, elevated serum triglycerides, and decreased high-density lipoprotein cholesterol concentrations. It is estimated to affect 1 in 3 people around the globe and is reported to affect 46% of surgical patients. For people with MetS who undergo surgery, an emerging body of literature points to significantly poorer postoperative outcomes compared with nonaffected populations. The aim of this study is to review the current evidence on the risks of surgical complications in patients with MetS compared to those without MetS.

**Methods:**

Systematic review and meta-analysis using PRISMA and AMSTAR reporting guidelines.

**Results:**

The meta-analysis included 63 studies involving 1 919 347 patients with MetS and 11 248 114 patients without MetS. Compared to individuals without the condition, individuals with MetS were at an increased risk of mortality (OR 1.75 95% CI: 1.36–2.24; *P*<0.01); all surgical site infection types as well as dehiscence (OR 1.64 95% CI: 1.52–1.77; *P*<0.01); cardiovascular complications (OR 1.56 95% CI: 1.41–1.73; *P*<0.01) including myocardial infarction, stroke, cardiac arrest, cardiac arrythmias and deep vein thrombosis; increased length of hospital stay (MD 0.65 95% CI: 0.39–0.9; *P*<0.01); and hospital readmission (OR 1.55 95% CI: 1.41–1.71; *P*<0.01).

**Conclusion:**

MetS is associated with a significantly increased risk of surgical complications including mortality, surgical site infection, cardiovascular complications, increased length of stay, and hospital readmission. Despite these risks and the high prevalence of MetS in surgical populations there is a lack of evidence on interventions for reducing surgical complications in patients with MetS. The authors suggest prioritising interventions across the surgical continuum that include (1) preoperative screening for MetS; (2) surgical prehabilitation; (3) intraoperative monitoring and management; and (4) postoperative rehabilitation and follow-up.

## Background

Highlights

- This meta-analysis involving 13 million individuals from various countries provided evidence that metabolic syndrome (MetS) was associated with a moderately increased risk of surgical complications.
- MetS was associated with an increased risk of adverse events including death, cardiovascular complications, surgical site infections, and hospital readmission.
- Our findings suggest that there is a need to implement screening processes for MetS prior to surgery, alert the surgical team to risks associated with MetS.

Metabolic syndrome (MetS) is a health condition characterised by a cluster of physiological and biochemical conditions that heighten the risk of adverse health outcomes^1–3^. Although some variations exist in specific diagnostic criteria, consensus statements by the WHO, a Joint Interim Statement (JIS) by prominent health organisations, and the National Cholesterol Education Programme Adult Treatment Panel III (NCEP III) identify MetS as an accumulation of at least three of the following five components: insulin resistance, obesity, chronic hypertension, elevated serum triglycerides, and decreased high-density lipoprotein cholesterol concentrations^2,3^.

It is important to understand the impact of MetS on surgical outcomes. Although some studies have shown no association between MetS and an increased risk of surgical complications^4,5^, there is a growing body of evidence suggesting those with the condition are at a greater risk of a range of serious adverse events during and after surgery^6–12^. Consequently, the costs of treating surgical patients with MetS are increased^5,13^. Evidence further suggests the accumulation of MetS components in individuals potentiates the risk of surgical complications compared to individual risk factors such as obesity^6^. For example, one study reported that patients with MetS have a higher rate of complications in bariatric surgery; in effect, this shows that patients undergoing bariatric surgery with MetS have a higher risk than those who have obesity alone^7^.

Despite the quantum of literature investigating the effect of MetS on surgical outcomes, no systematic review and meta-analysis of the evidence has been performed to date. Moreover, to our knowledge, there are no reported interventions or guidelines in the literature on ameliorating the risks associated with MetS. There is a need for quality appraisal and synthesis of the accumulated evidence to identify whether MetS predisposes patients to a greater risk during surgery than those without MetS. The aim of this review is to therefore synthesise the evidence on the risks of surgical complications in patients with MetS compared to those without MetS.

## Method

**Figure 1:**
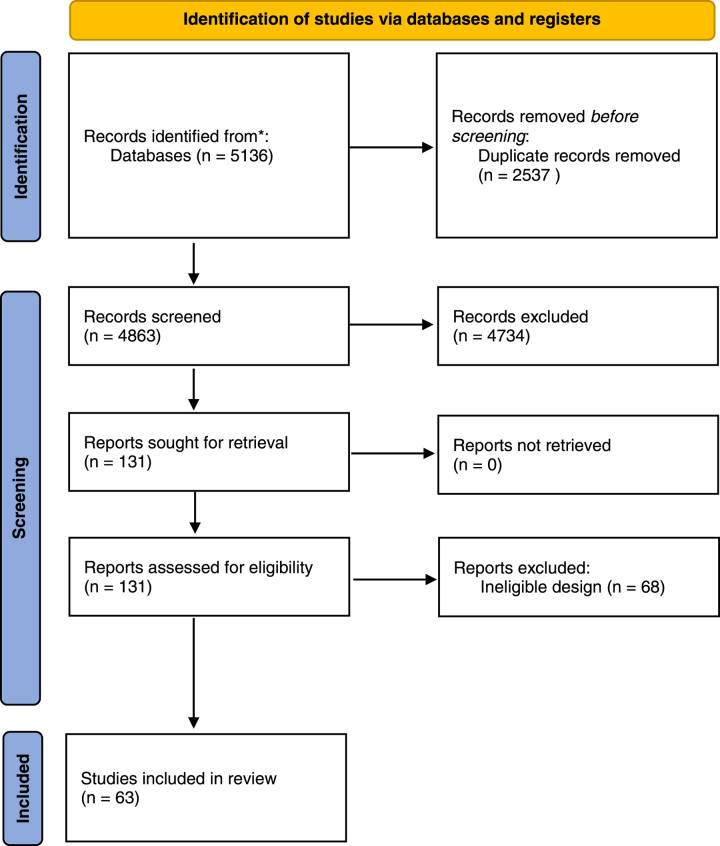
PRISMA flowchart.

We conducted a systematic literature search according to the Preferred Reporting Items for Systematic Reviews and Meta-Analyses (PRISMA) recommendations (see Fig. 1) and in compliance with Assessing the Methodological Quality of Systematic Reviews (AMSTAR) guidelines^8,9^. A review protocol was registered *a priori* with PROSPERO (BLINDED) and also researchregistry.com (reviewregistry1703). The review protocol is published elsewhere (BLINDED).

### Search strategy

We used the database search approach recommended by Bramer and colleagues^10^ searching: CINAHL, Embase, Google Scholar (Top 200), PsycINFO, PubMed, and Web of Science. Database searches were supplemented by backward and forward citation tracking of included articles using Scopus. A date limitation of greater than or equal to 1998 was applied to the search strategy to reflect the first formal definition of MetS^2^. The full search strategy is provided (see Table 1).

**Table 1:** Search Strategy.

Database	Search Strategy
PubMed	(((“metabolic syndrome”[MeSH Major Topic]) OR (“metabolic syndrome”[Title/Abstract])) OR (“deadly quartet”[Title/Abstract])) AND (((((((((“surgery”[Title/Abstract]) OR (“surgical”[Title/Abstract])) OR (“perioperative”[Title/Abstract])) OR (“preoperative”[Title/Abstract])) OR (“intraoperative”[Title/Abstract])) OR (“postoperative”[Title/Abstract])) OR (“intraoperative complications”[MeSH Major Topic])) OR (“postoperative complications”[MeSH Major Topic])) OR (“surgical procedures, operative”[MeSH Major Topic])) Filters: English, Humans, from 1998 to 2022
CINAHL	(S1 AND S2) S1 MH metabolic syndrome OR TI deadly quartet OR AB deadly quartet OR TI metabolic syndrome OR AB metabolic syndrome S2 TI surgery OR AB surgery OR TI preoperative OR AB preoperative OR TI postoperative OR AB postoperative OR TI intraoperative OR AB intraoperative OR MH postoperative complications OR MH surgical procedures, operative OR MH intraoperative complications Limiters - Published Date: 19980101-20221231 Expanders - Apply equivalent subjects Narrow by Language: - english Search modes - Boolean/Phrase
PsycINFO	(S1 AND S2) S1 MH metabolic syndrome OR TI deadly quartet OR AB deadly quartet OR TI metabolic syndrome OR AB metabolic syndrome S2 TI surgery OR AB surgery OR TI preoperative OR AB preoperative OR TI postoperative OR AB postoperative OR TI intraoperative OR AB intraoperative OR MH postoperative complications OR MH surgical procedures, operative OR MH intraoperative complications Limiters - Published Date: 19980101-20221231 Expanders - Apply equivalent subjects Narrow by Language: - english Search modes - Boolean/Phrase
Google Scholar	“metabolic syndrome” OR “deadly quartet” AND surgery OR surgical OR perioperative OR preoperative OR intraoperative OR postoperative
Web of Science	surgery (Title) or surgery (Abstract) or surgical (Title) or surgical (Abstract) or perioperative (Title) or perioperative (Abstract) or preoperative (Title) or preoperative (Abstract) or intraoperative (Title) or intraoperative (Abstract) or postoperative (Title) or postoperative (Abstract) AND metabolic syndrome (Title) or metabolic syndrome (Abstract) or deadly quartet (Title) or deadly quartet (Abstract)
Embase	1. metabolic syndrome.ab. or metabolic syndrome.ti. or deadly quartet.ab. or deadly quartet.ti. 2. surgery.ab. or surgery.ti. or surgical.ab. or surgical.ti. or perioperative.ab. or perioperative.ti. or intraoperative.ab. or intraoperative.ti. or preoperative.ab. or preoperative.ti. or postoperative.ab. or postoperative.ti. 3. 1 and 2 4. limit 3 to (full-text and human and english) 5. limit 4 to yr=“1998 - Current” 6. limit 5 to (full-text and human and english language) 7. limit 6 to ((embase or “preprints (unpublished, non-peer-reviewed)”) and journal)

### Eligibility criteria

We included published peer-reviewed studies that reported on the effect of MetS on the occurrence of surgical complications in adult patients undergoing invasive surgery. Studies were included if they were prospective or retrospective observational studies that reported on 30-day complications in adult surgical patients diagnosed with MetS. As the criteria to establish a diagnosis of MetS may vary, we accepted the definition of MetS as defined by the study authors. Studies were excluded if they reported on surgical complications greater than 30 days or minor surgical procedures (e.g. lesion removal) (see Table 2. Inclusion and Exclusion Criteria).

**Table 2:** Inclusion and exclusion criteria.

Inclusion Criteria	
Observational studies (e.g. cohort study) Adult human patients (18 years or >) Undergoing invasive surgery^*^	Diagnosed with metabolic syndrome Complications within 30 days of surgery Published peer-reviewed articles
Exclusion Criteria	
Publication Type Narrative reviews Editorials Government reports Books or book chapters Conference proceedings Commentaries Lectures and presentations	Study Design Interventional studies Studies not included in the meta-analysis Systematic Reviews Study Population Animals Children Study Procedure Minor procedures (e.g. lesion removal; cystoscopy;) Complications >30 days after surgery

* For the purposes of this study, invasive surgery was considered any surgical procedure involving a skin incision and surgical dissection below the level of the dermis (excludes skin excisions, biopsy etc.) and/or instrumentation of a natural orifice in conjunction with an excisional procedure (urology, gynaecology, etc.).

### Study selection

Following the initial search for studies, citations were exported into EndNote software^11^.

After the removal of duplicates, the title and abstracts of studies were screened by two independent reviewers (P.N. and N.R.) against inclusion and exclusion criteria to identify studies for potential inclusion. The full-text of each selected article was screened by two independent reviewers (P.N. and N.R.) to determine eligibility against the inclusion and exclusion criteria. Disagreement consensus was achieved through discussion between reviewers (P.N. and N.R.).

### Data management

One review author (P.N.) extracted data from the included studies using a preconstructed data extraction form. Authors were contacted in instances of missing or ambiguous data. Studies were excluded where the author did not respond, or data extraction was not possible. Extracted data was entered into Review Manager (RevMan) Version 5.4.1^12^, which another review author (N.R.) independently checked for accuracy.

### Data extraction

Outcomes of interest were the risk of complications within 30 days of surgery, length of stay (LOS), and hospital readmission. We accepted the definition for each surgical complication provided by the authors of each included study and extracted data on 30-day surgical complications. Outcomes included were mortality, surgical site infection (SSI) (any SSI, superficial SSI, deep SSI, organ space SSI, and dehiscence), cardiovascular complications (arrhythmia, myocardial infarction (MI), cardiac arrest, stroke, deep vein thrombosis (DVT), LOS, and hospital readmission. For categorical data, the number of events in the control, and exposure cohorts were extracted. For continuous data, the mean difference (MD) and SD values were extracted.

### Statistical analysis

Meta-analysis was performed using Review Manager (RevMan 5.4.1)^12^. The effect estimate with a 95% CI were extracted from each included study. We extracted the effect size reflecting the greatest degree of adjustment for possible confounding factors when multiple effect sizes with different degrees of covariate adjustment were reported in a study. For continuous variables, to estimate the summary effect size we used a random-effects model and the inverse-variance method to obtain MDs and SDs with 95% CIs. For dichotomous variables, the Mantel–Haenszel formula was used to produce a single summary measure of association to obtain odds ratios (ORs) along with its CIs. We used a random-effects model for pooled analysis regardless of heterogeneity since this model estimates the effect with consideration to the variance between studies, rather than ignoring heterogeneity by employing a fixed effect model^14^. Heterogeneity of studies was estimated using the Higgins *I*^2^ statistic^15^ and described as low (25%), moderate (25–55%), and high (>75%)^16^. The *P*-value for statistical significance was set at ≤0.05. We removed one study at a time to observe the effect on the results. We then calculated an overall estimate of effect size using a random-effects meta-analysis based on the adjusted OR of all included studies.

### Quality assessment

Two independent reviewers (P.N. and N.R.) performed quality assessment. Each included study was critically appraised using the Newcastle–Ottawa Scale (NOS) for observational studies. The NOS is a widely used and endorsed scale to assess the quality of observational studies^17,18^. The NOS is validated for assessing three quality parameters, namely, selection, comparability, and outcome divided across eight specific items. Studies were independently screened and scored (0–9) by two reviewers (P.N. and N.R.). Each study was assessed against criteria and scored according to good (7–9), fair (4–6), and poor quality (<4). Discrepancy in assessment scores were resolved through discussion and consensus (see Table 3).

**Table 3:** Table of included studies.

References	Year	Country	Study Design	Sample	Surgery Type	NOS Score	NOS Quality Rating
Angeloni *et al*.^19^	2012	Italy	Retrospective analysis	1726	Cardiac	8	Good
Ardeshiri^20^	2014	Iran	Prospective analysis	235	Cardiac	8	Good
Arnaoutakis *et al*.^4^	2014	USA	Retrospective analysis	19 604	Vascular	8	Good
Aydogan *et al*.^21^	2019	Turkey	Prospective analysis	120	Urology	8	Good
Bacalbasa *et al*.^22^	2020	Romania	Retrospective analysis	46	Gynaecology	7	Good
Bhayani *et al*.^23^	2012	USA	Retrospective analysis	3973	Hepatobiliary	7	Good
Casana *et al*.^24^	2019	Italy	Retrospective analysis	752	Vascular	8	Good
Chen *et al*.^25^	2020	China	Prospective analysis	628	Gastrointestinal surgery	8	Good
Chung *et al*.^26^	2018	USA	Retrospective analysis	15 618	Orthopaedic	8	Good
Cichos *et al*.^27^	2018	USA	Retrospective analysis	3 348 207	Orthopaedic	8	Good
Doyle *et al*.^28^	2017	Ireland	Prospective analysis	113	Gastrointestinal surgery	8	Good
Edelstein^29^	2016	USA	Retrospective analysis	1462	Orthopaedic	7	Good
Edelstein *et al*.^30^	2017	USA	Retrospective analysis	107 117	Orthopaedic	7	Good
Elsamna *et al*.^31^	2020	USA	Retrospective analysis	41 788	Emergency general surgery	8	Good
Elsamna *et al*.^32^	2021	USA	Retrospective analysis	138 318	Endocrine surgery	8	Good
Fagenson *et al*.^33^	2021	USA	Retrospective analysis	1726	Hepatobiliary	7	Good
Garcia *et al*.^15^	2016	USA	Retrospective analysis	4753	Orthopaedic	8	Good
Gazivoda *et al*.^34^	2022	USA	Retrospective analysis	19 054	Hepatobiliary	8	Good
Goshtasbi *et al*.^35^	2022	USA	Retrospective analysis	52 261	ENT	8	Good
Hobeika *et al*.^36^	2019	France	Retrospective analysis	115	Hepatobiliary	8	Good
Hong *et al*.^37^	2010	Republic of Korea	Retrospective analysis	740	Cardiac	8	Good
Hudetz *et al*.^38^	2011	USA	Prospective analysis	56	Cardiac	7	Good
Inabnet *et al*.^39^	2012	USA	Retrospective analysis	186 576	Bariatric	8	Good
Jehan *et al*.^40^	2020	USA	Retrospective analysis	4572	Gastrointestinal surgery	8	Good
Kajimoto *et al*.^41^	2009	Japan	Retrospective analysis	1183	Cardiac	8	Good
Kunt *et al*.^42^	2016	Turkey	Retrospective analysis	494	Cardiac	6	Fair
Lak *et al*.^43^	2019	USA	Retrospective analysis	59 404	Bariatric	8	Good
Laou *et al*.^44^	2017	Greece	Prospective analysis	105	Hepatobiliary	7	Good
Lohsiriwat *et al*.^45^	2010	Thailand	Prospective analysis	114	Colorectal	8	Good
Lovecchio *et al*.^6^	2018	USA	Retrospective analysis	18 605	Orthopaedic	8	Good
Malik *et al*.^46^	2019	USA	Retrospective analysis	15 735	Orthopaedic	8	Good
Malik *et al*.^47^	2019	USA	Retrospective analysis	31 621	Orthopaedic	8	Good
Memtsoudis^5^	2012	USA	Retrospective analysis	238 296	Orthopaedic	8	Good
Menendez^48^	2014	USA	Retrospective analysis	669 841	Orthopaedic	8	Good
Nia *et al*.^49^	2019	USA	Retrospective analysis	15 136	Neurosurgery	8	Good
Özkan *et al*.^50^	2017	Turkey	Prospective analysis	152	Cardiac	8	Good
Ozyazicioglu^51^	2010	Turkey	Prospective analysis	83	Cardiac	5	Fair
Panayi *et al*.^52^	2022	USA	Retrospective analysis	3809	Plastic surgery	8	Good
Pertsch *et al*.^53^	2022	USA	Retrospective analysis	14 310	Vascular	8	Good
Pimenta^54^	2007	Brazil	Prospective analysis	107	Cardiac	6	Fair
Raviv *et al*.^55^	2017	USA	Retrospective analysis	47 386	Hepatobiliary	7	Good
Riddle *et al*.^56^	2020	USA	Retrospective analysis	12 827	Plastic surgery	8	Good
Sarna *et al*.^57^	2022	USA	Retrospective analysis	670 935	Bariatric	8	Good
Selph *et al*.^58^	2014	USA	Retrospective analysis	11 865	Urologic	7	Good
Shariq *et al*.^59^	2018	USA	Retrospective analysis	3502	Endocrine	8	Good
Shariq *et al*.^60^	2019	USA	Retrospective analysis	91 566	Colorectal	8	Good
Smolock *et al*.^61^	2012	USA	Retrospective analysis	739	Vascular	7	Good
Sorber *et al*.^62^	2019	USA	Retrospective analysis	10 053	Vascular	8	Good
Swart *et al*.^63^	2012	South Africa	Retrospective analysis	873	Cardiac	6	Fair
Tadic *et al*.^64^	2014	Serbia	Retrospective analysis	182	Cardiac	8	Good
Tanaka *et al*.^65^	2018	USA	Retrospective analysis	154	Vascular	8	Good
Tee *et al*.^66^	2016	USA	Retrospective analysis	15 831	Hepatobiliary	8	Good
Tracey *et al*.^67^	2022	USA	Retrospective analysis	37 495	Orthopaedic	8	Good
Visser *et al*.^68^	2017	Netherlands	Retrospective analysis	564	Vascular	8	Good
Wang *et al*.^69^	2018	China	Retrospective analysis	1166	Cardiac	8	Good
Williams *et al*.^70^	2014	USA	Retrospective analysis	79	Vascular	7	Good
Wu *et al*.^71^	2022	China	Prospective analysis	585	Gastrointestinal	8	Good
Xiaoqi *et al*.^72^	2020	China	Retrospective analysis	2880	Orthopaedic	6	Fair
Xie *et al*.^73^	2020	USA	Retrospective analysis	15 069	Orthopaedic	7	Good
Xu^74^	2019	China	Retrospective analysis	606	Urology	8	Good
Ye *et al*.^75^	2020	USA	Retrospective analysis	6696	Orthopaedic	8	Good
Zapata *et al*.^76^	2020	USA	Retrospective analysis	11 020	Cardiac	8	Good
Zavlin *et al*.^77^	2017	USA	Retrospective analysis	7030	Plastic surgery	8	Good

## Results

### Study selection and characteristics

In total, 4863 abstracts were reviewed, from which 131 full-text articles were retrieved and evaluated for inclusion (see Fig. 1). Sixty-three studies, involving 1 919 347 patients with MetS and 11 248 114 patients without MetS, satisfied the inclusion criteria (see Table 2. Inclusion and Exclusion Criteria). The most commonly reported types of surgery were orthopaedic (22%)^5,6,15,26,27,29,46–48,67,72,73,75,78^, cardiac (21%)^19,20,37,38,41,42,50,51,54,63,64,69,76^, vascular (13%)^4,24,53,62,65,68,70,79^, hepatobiliary (11%)^50–56^, gastroenterology (6%)^57–60^, bariatric (5%)^39,43,57^, urology (5%)^21,58,74^, and plastics (5%)^52,56,77^ (see Table 3. Included Studies). Most studies reported North America data (*n*=40)^4–6,15,23,27,30–35,38–40,43,46–49,52,53,55–62,65–67,70,73,75–78,80^, with the remaining spanning Europe^19,21,22,24,28,42,44,50,51,64,68,81^, the Middle East^20^, Asia^37,41,45,71,72,74,82,83^, Africa^63^, and South America^54^. The assessed risk of bias of the included studies ranged from 5 to 8 (fair to good) out of a possible 9 stars when assessed using the NOS.

### Mortality

**Figure 2:**
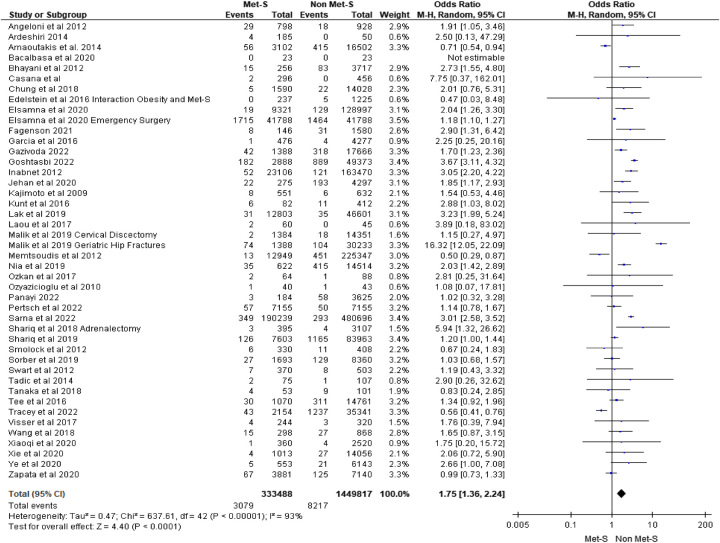
30-day mortality.

Mortality within 30 days of surgery was reported in 44 studies considered for meta-analysis. Across these studies, a total of 333 488 patients with MetS underwent surgery versus 1 449 817 surgical patients without MetS. We found fifteen of 44 studies reported an increased risk of mortality across a range of surgical types including bariatric^39,43,57^, cardiac^19,42^, ear, nose, and throat (ENT)^35^, emergency^31^, endocrine^59,84^, gastrointestinal^40^, hepatobiliary^23,33,34^, neurosurgery^49^, and orthopaedic^47^. Twenty-five studies considered for meta-analysis found no association with 30-day mortality between MetS and non-MetS patients across a wide range of surgical types. Three studies focusing on orthopaedic and vascular surgical patients reported those with MetS were at less risk of 30-day mortality^4,5,67^. One study focused on gynaecological surgery reported no deaths in either group between the MetS and non-MetS groups^22^. On pooling of effect estimates, surgical patients with MetS were at 1.75 times the risk of death within 30 days after surgery compared to patients without MetS (OR 1.75 95% CI: 1.36–2.24; *P*<0.0001) (see Fig. 2).

### Cardiovascular events

#### MI

**Figure 3:**
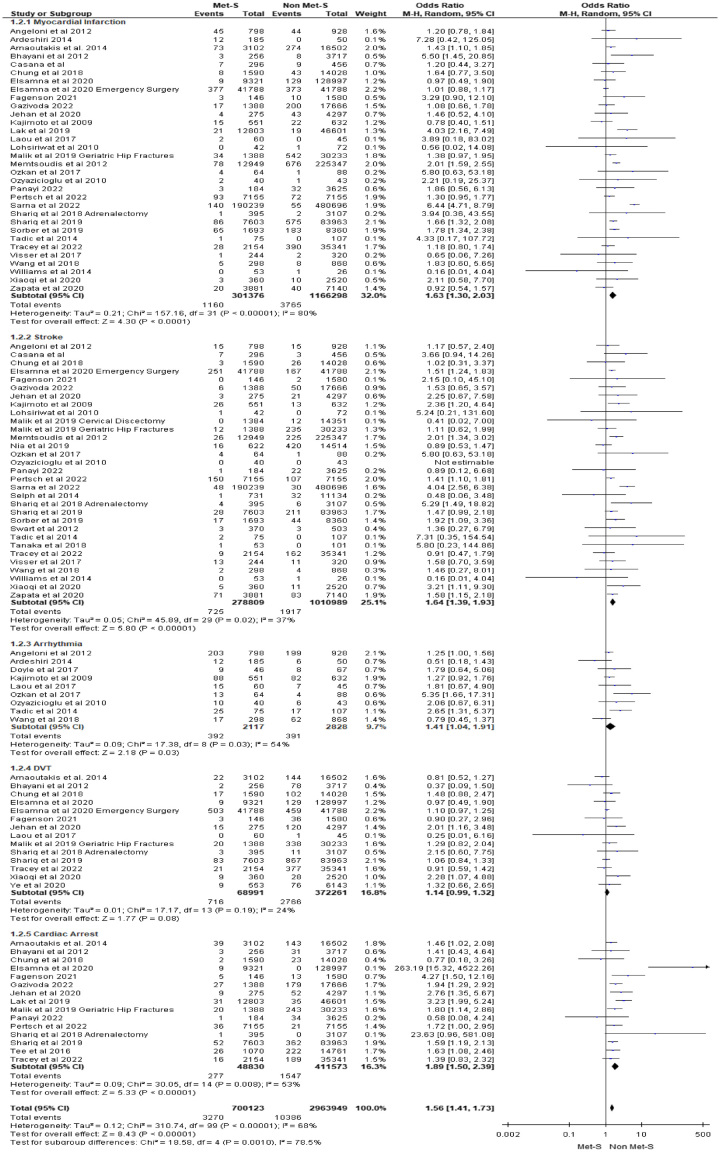
30-day cardiovascular complications.

MI within 30 days of surgery was reported in 32 studies included for meta-analysis. Across these studies, a total of 301 376 patients with MetS underwent surgery versus 116 6298 without MetS. We found 7 of 32 studies reported an increased risk of MI across a range of surgeries including bariatric^43,57^, colorectal^60^, hepatobiliary^23^, orthopaedics^5^, and vascular^4,62^. Twenty-five studies reported no association between 30-day operative MI and patients with or without MetS. Meta-analysis of studies revealed surgical patients with MetS were at 1.63 times the risk of MI within 30 days after surgery compared to patients without MetS (OR 1.63 95% CI: 1.30–2.03; *P*=0.001) (see Fig. 3 for all Cardiovascular Events).

#### Stroke

Stroke was reported in 31 studies included for meta-analysis. Across these studies, a total of 278 809 patients with MetS underwent surgery versus 1 010 989 surgical patients without MetS. We identified that 9 out of 31 studies reported an increased risk of stroke across a range of surgeries including bariatric^57^, cardiac^41,76^, emergency general surgery^31^, endocrine^59^, orthopaedic^5^ and vascular^53,62^. Twenty-one studies reported no association with 30-day stroke between MetS and non-MetS patients across a range of surgeries. No strokes occurred in a single study^85^. The link between MetS and stroke within 30 days of surgery was identified following a meta-analysis. Individuals with MetS were at 1.64 times the risk of stroke within 30 days after surgery compared to patients without MetS (OR 1.64 95% CI: 1.39–1.93]; *P*=0.00001).

#### Cardiac arrhythmias

Cardiac arrhythmias within 30 days of surgery were reported in nine studies included for meta-analysis with a total sample of 2117 patients with MetS versus 2828 surgical patients without MetS. In the studies considered for meta-analysis, 2 out of 9 studies reported an increased risk of cardiac arrhythmias during cardiac surgery^50,64^, while the remaining seven studies identified no association with 30-day cardiac arrhythmias between MetS and non-MetS patients across a range of surgeries. Pooling of effect estimates revealed surgical patients with MetS were at 1.41 times the risk of cardiac arrhythmias within 30 days after surgery compared to patients without MetS (OR 1.41 95% CI: 1.04–1.91; *P*=0.03).

#### DVT

DVT within 30 days of surgery was reported in 14 studies included for meta-analysis. Across these studies, a total of 68 991 patients with MetS underwent surgery versus 372 261 non-MetS patients. Two of 14 studies reported an increased risk of DVT across gastroenterology^40^ and orthopaedic surgeries^72^. The remaining 12 studies included for meta-analysis found no association with 30-day DVT presentations in MetS and non-MetS patients across a range of surgeries. The link between MetS and DVT within 30 days of surgery was not identified following a meta-analysis. Pooling of effect estimates revealed surgical patients with MetS were at 1.14 times the risk of 30-day DVT compared to patients without MetS, but statistical significance was not reached (OR 1.14 95% CI: 0.99–1.32; *P*=0.08).

#### Cardiac arrest

Cardiac arrest within 30 days of surgery was reported in 15 of the included studies. Across these studies, a total of 48 830 patients with MetS underwent surgery versus 411 573 patients without MetS. Ten of 15 studies reported an increased risk of cardiac arrest across bariatric^43^, colorectal^60^, endocrine^59^ gastroenterology^40^, hepatobiliary^33,34,66^, orthopaedic^47^, and vascular surgeries^4,53^. The remaining five studies included for meta-analysis found no association with cardiac arrest within 30 days of surgery. Pooling of effect estimates revealed surgical patients with MetS were at 1.89 times the risk of cardiac arrest compared to patients without MetS, (OR 1.89 95% CI: 1.5–2.39; *P*<0.00001).

#### Grouped cardiovascular complications

A total of 40 of 63 studies reported a grouped outcome of cardiovascular complications that were not categorised by specific type within 30 days of surgery comprising 700 123 patients with MetS versus 2 963 949 surgical patients without MetS. Surgical patients with MetS were at 1.56 times the risk of any cardiovascular complication within 30 days after surgery compared to patients without MetS (OR 1.56 95% CI: 1.41–1.73; *P*=0.00001).

### SSIs

#### Superficial SSI

**Figure 4:**
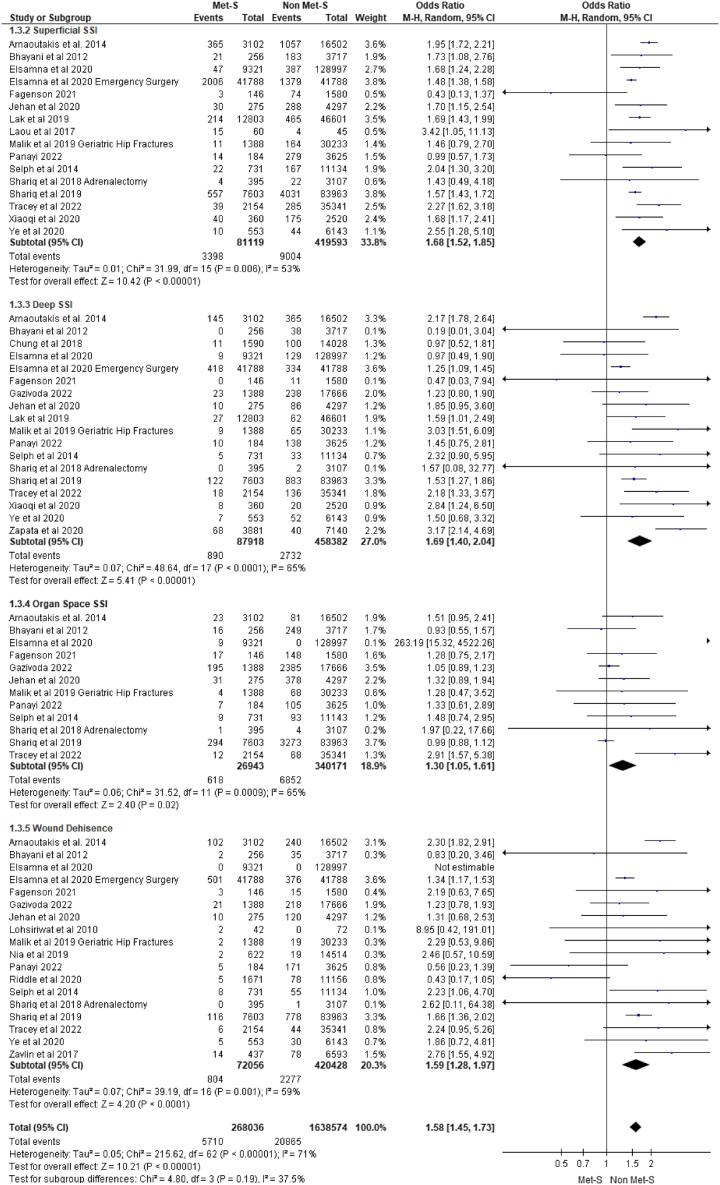
Surgical site infections.

Superficial SSIs within 30 days of surgery were reported in 16 studies. A total of 81 119 patients with MetS underwent surgery versus 419 593 surgical patients without MetS. Twelve studies reported an increased risk of superficial SSI across a range of surgeries including bariatric^43^, colorectal^60^, emergency general surgery^31^, endocrine^32^, gastroenterology^40^, hepatobiliary^23,44^, orthopaedic^67,72,75^, urologic^58^, and vascular^4^ while the remaining four studies found no association. Pooling of effect estimates revealed surgical patients with MetS were at 1.68 times the risk of 30-day superficial SSI compared to patients without MetS (OR 1.68 95% CI: 1.52–1.85; *P*=0.01) (see Fig. 4 for all SSI data).

#### Deep SSI

Deep SSIs within 30 days of surgery were reported in 18 studies. Across these studies, a total of 87 918 patients with MetS underwent surgery versus 458 382 surgical patients without MetS. In eight included studies, an increase in deep SSI was observed across a range of surgeries including bariatric^43^, cardiac^76^, colorectal^60^ emergency general surgery^31^, orthopaedic^47,67,72^, and vascular^4^. The remaining 10 studies included for meta-analysis reported no association with 30-day deep SSI. Pooling of effect estimates revealed surgical patients with MetS were at 1.69 times the risk of 30-day deep SSI compared to patients without MetS, (OR 1.69 95% CI: 1.40–2.04; *P*=0.00001).

#### Organ space SSI

The incidence of organ space SSIs within 30 days of surgery was reported in 12 studies. Across these studies, a total of 26 943 patients with MetS underwent surgery versus 340 171 surgical patients without MetS and were monitored for organ space SSIs. In 2 of 12 studies, an increase was reported in organ space SSIs across endocrine^84^ and orthopaedic^67^, specialities while 10 studies found no association. Pooling of effect estimates revealed surgical patients with MetS were at 1.3 times the risk of organ space SSIs within 30 days of surgery compared to patients without MetS, (OR 1.3 95% CI: 1.05–1.61; *P*=0.02).

#### Dehiscence

Wound dehiscence within 30 days of surgery was reported in 18 studies. Across these studies, a total of 72 056 patients with MetS underwent surgery versus 420 428 surgical patients without MetS. In five studies, an increase in wound dehiscence was observed across a range of surgeries including colorectal^60^, emergency general surgery^31^, plastics^77^, urologic^58^, and vascular^4^. Twelve studies included for meta-analysis found no association with 30-day wound dehiscence between MetS and non-MetS patients. In a single study^32^ where wound dehiscence was an outcome, none were observed. Pooling of effect estimates revealed surgical patients with MetS were at 1.59 times the risk of 30-day wound dehiscence compared to patients without MetS, (OR 1.59 95% CI: 1.28–1.97; *P*=0.0001).

#### Uncategorised SSI

A total of 40 of the 63 studies reported SSIs; however, did not provide a classification according to standardised definitions. Across these studies, a total of 477 207 patients with MetS underwent surgery versus 2 295 152 surgical patients without MetS and were monitored for an SSI occurring within 30 days of surgery. Surgical patients with MetS were at 1.64 times the risk of an uncategorised SSI within 30 days after surgery compared to patients without MetS (OR 1.64 95% CI: 1.52–1.77; *P*=0.00001).

### Hospital readmission

**Figure 5:**
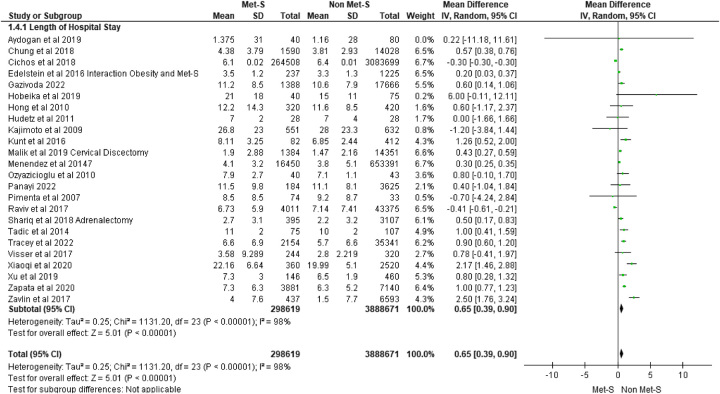
Hospital length of stay.

Hospital readmission within 30-day of surgery was reported in 22 studies included for meta-analysis. Across these studies, a total of 109 910 patients with MetS underwent surgery versus 650 525 surgical patients without MetS. In the studies considered for meta-analysis, 14 out of 22 were statistically associated with an increase in hospital readmission across a range of surgeries including bariatric^39^, endocrine^32,59^, ENT^35^, emergency general surgery^31^, gastrointestinal^40^, orthopaedic^6,46,47,67,73,78^, and plastics^56,77^. The remaining eight studies included for meta-analysis found no association with hospital readmission between MetS and non-MetS patients. Pooling of effect estimates revealed surgical patients with MetS were at 1.55 times the risk of hospital readmission compared to patients without MetS, (OR 1.55 95% CI: 1.41–1.71; *P*=0.00001) (see Fig. 5).

### Hospital LOS

**Figure 6:**
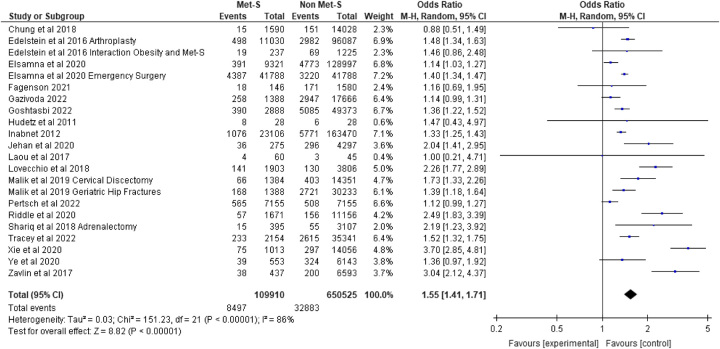
Readmission.

Hospital LOS was reported in 24 included studies. Across these studies, a total of 298 619 patients with MetS underwent surgery versus 3 888 671 surgical patients without MetS. In 13 of 24 studies, an increased length of hospital stay was reported across a range of surgeries including cardiac^42,64,76^, endocrine^59^, hepatobiliary^34^, orthopaedic^26,48,67,72,78,86^, plastics^77^, and urology^74^. Two studies focusing on orthopaedic and hepatobiliary surgical patients^27,55^ reported those with MetS were at less risk of increased hospital LOS. A further nine studies found no association with the length of hospital stay between MetS and non-MetS patients across a range of surgeries. Pooling of effect estimates revealed surgical patients with MetS experienced an increased length of hospital stay (MD 0.65 95% CI: 0.39–0.9; *P*=0.00001) (see Fig. 6).

## Discussion

This review of 13 167 461 participants across 63 included studies demonstrates that patients with MetS undergoing surgery are at an increased risk of adverse outcomes within 30 days postoperatively. While components of MetS (insulin resistance, obesity, chronic hypertension, elevated serum triglycerides, and decreased high-density lipoprotein)^87–90^ are known to be independent risk factors for adverse surgical outcomes, our meta-analysis demonstrates an increased risk of adverse outcomes where these risk factors accumulate to meet the MetS diagnostic criteria. Our review indicates that where surgical patients are identified with MetS, they have a 75% increased risk of death; a 56% increased risk of cardiovascular complications; a twofold increased risk of any SSI; and a 55% increased risk of hospital readmission. Considering MetS is both highly prevalent and associated with an increased likelihood of adverse complications after surgery, our findings indicate the need to (1) identify MetS in surgical patients using evidence-based screening approaches, and (2) implement guidelines that treat relevant components of MetS at optimal time points around surgery.

Adopting standardised diagnostic criteria for MetS could facilitate improved detection and the initiation of management strategies throughout the surgical continuum to improve patient outcomes. For instance, most routine preoperative assessments are likely to include assessments that record NCEP III diagnostic criteria of insulin resistance, obesity, chronic hypertension, elevated serum triglycerides, and decreased high-density lipoprotein. Hospital systems should incorporate alerts where a patient meets the diagnostic criteria for MetS as part of existing presurgical screening processes to allow better detection of this patient cohort and identification of the risks associated with a diagnosis of MetS prior to surgery. Identifying these risks is important as it is well established that there is an additive effect of risk factors on short-term and long-term surgical outcomes that can be demonstrated using surgical risk calculators such as the ACS-NSQIP and CeDAR^91^. Based on the results of our review, it is likely that the surgical risks imposed by MetS criteria are also additive in nature and should be incorporated into existing surgical risk calculators to provide a more comprehensive assessment of the risk profile of this patient cohort. Furthermore, it is important to impart awareness of the risk to patients with MetS as part of the consent process. This conversation needs to occur contemporaneously with efforts to minimise communication bias, discrimination, and weight stigmatisation^92^. Treating MetS effectively may necessitate delaying elective surgery or implementing an enhanced recovery after surgery protocol, which in turn, may increase patient frustration, anxiety and challenges with surgical optimisation.

Healthcare providers should therefore take steps to implement prehabilitative, intraoperative, and rehabilitative approaches to care to improve patient recovery, facilitate earlier discharge from the hospital, and potentially reduce healthcare costs by lowering or eliminating complications associated with MetS including hospital readmission. Surgical optimisation interventions have shown promise for some of the diagnostic criteria of MetS such as treating obesity^93^ and hypertension^94^ prior to surgery, thus, there is the potential to adapt elements of existing interventions and guidelines for MetS patients to eliminate or reduce operative risks. It is also vital that the surgical team is attentive to practices that reduce SSI including weight-based dosing of prophylactic antibiotics^95^ , redosing of prophylactic antibiotics in longer operations^96^, glucose optimisation^97^, glove changes^98^, and the use of alcoholic skin preparation prior to skin closure^99^. Postoperative follow-up and rehabilitation of these patients should also emphasise initiatives that reduce the risk of potential postoperative complications, such as SSIs^100,101^, venous thromboembolism events^102,103^, and cardiovascular complications^104,105^.

One limitation of this review stems from the varying definitions of MetS used in the included studies, which can lead to population heterogeneity and complicate result comparisons. Additionally, the inclusion of retrospective observational studies, drawing data from medical records databases, introduces potential biases and limitations, including incomplete information, selection bias, and possible confounding factors. In summation, the review provides valuable insights and is the largest review of the surgical risks patients with MetS face. It also provides socio-ecological validity by drawing evidence globally from countries with similarly developed health systems and highlights a significant risk profile which, heretofore, has not been addressed with review level evidence.

## Conclusion

Our review is the largest, most-comprehensive analysis of postoperative surgical complications in MetS. Our findings highlight that surgical patients with MetS are at a heightened risk of a range of adverse outcomes in the 30 days following surgery. Based on our findings, firstly, there is a need to implement evidence-based screening approaches to identify MetS in surgical patients to facilitate early detection and initiate management strategies prior to, during, and after surgery for improved outcomes. Secondly, the surgical team must be aware of the increased risks associated with MetS, be alerted to a diagnosis preoperatively, communicate risks to the patient during the consent process, and treat components of the condition to avoid the risks of adverse events. In conclusion, early detection, personalised management, and comprehensive perioperative care for MetS patients are essential to mitigate risks, enhance outcomes, and potentially reduce healthcare costs by minimising complications and readmissions.

## Ethical approval

Not applicable.

## Patient consent

Not applicable.

## Sources of funding

PN is supported by an Australian Government research scholarship.

## Conflicts of interest disclosure

The authors declare that they have no financial conflict of interest with regard to the content of this report.

## Author contribution

P.N. and N.R.: devised the review concept, developed the protocol, conducted the search strategy, data extraction, analysis of the results, and draughted the review; A.C., F.F., T.A., and J.G.: contributed to the review and authoring of further manuscript drafts.

## Research registration unique identifying number (UIN)

1. Name of the registry: researchregistry.com.
2. Unique identifying number or registration ID: researchregistry1703.
3. Hyperlink to your specific registration (must be publicly accessible and will be checked): https://www.researchregistry.com/browse-the-registry#registryofsystematicreviewsmeta-analyses/registryofsystematicreviewsmeta-analysesdetails/65079c75372670002503ab1b/.

## Guarantor

Philip Norris and Nicholas Ralph.

## Data availability statement

Data from the review is available on request.

## Provenance and peer review

This review is not commissioned and was submitted to IJS for external peer-review by blinded peer reviewers.
